# The relationship between proportions of carbohydrate and fat intake and hyperglycaemia risk in Chinese adults

**DOI:** 10.1017/S1368980024001204

**Published:** 2024-06-03

**Authors:** Yayun Fan, Qingqing Huang, Honglan Gao, Fengying Huang, Dingliu He

**Affiliations:** 1 Department of Clinical Nutrition, Yancheng Clinical College of Xuzhou Medical University, The First People’s Hospital of Yancheng, Yancheng, China; 2 Department of Clinical Laboratory, The First Affiliated Hospital of Anhui Medical University, Hefei, Anhui, China; 3 Department of Clinical Nutrition, Third Affiliated Hospital of Soochow University, Changzhou, Jiangsu, China

**Keywords:** China Health and Nutrition Survey, Proportions of carbohydrate and fat, Hyperglycaemia, High carbohydrate and low fat (HCLF), Low carbohydrate and high fat (LCHF)

## Abstract

**Objective:**

To address the relationship between the proportions of carbohydrates and fat and hyperglycaemia in the Chinese population.

**Design:**

A cross-section research involving data from the China Health and Nutrition Survey in 2009, and nutritional status and health indicators were mainly focused.

**Setting:**

China.

**Participants:**

8197 Chinese individuals aged over 16 years, including 1345 subjects who had a low-carbohydrate and high-fat diet, 3951 individuals who had a medium proportion of carbohydrate and fat diet, 2660 participants who had a high-carbohydrate and low-fat diet and 241 people who had a very-high-carbohydrate and low-fat diet.

**Results:**

Subjects with the high-carbohydrate and low-fat diet were significantly associated with an increased risk of hyperglycaemia (OR: 1·142; 95 % CI: 1·022, 1·276) when compared with the individuals with the medium proportion of carbohydrate and fat diet. Meanwhile, people with a very-high-carbohydrate and low-fat diet had a higher risk of hyperglycaemia (OR: 1·829; 95 % CI: 1·377, 2·429). In contrast, the association between participants with a low-carbohydrate and high-fat diet and hyperglycaemia was NS (OR: 1·082; 95 % CI: 0·942, 1·243) with adjusting a series of confounding factors. Furthermore, people with a very-high-carbohydrate and low-fat diet were significantly associated with a higher risk of hyperglycaemia in the major energy levels and social characteristics subgroup.

**Conclusions:**

We found the high-carbohydrate and low-fat and very-high-carbohydrate and low-fat diets were significantly associated with a high risk of hyperglycaemia. And, the association between low-carbohydrate and high-fat diets and the risk of hyperglycaemia was NS.

In recent years, with changes in people’s lifestyles and social progress, hyperglycaemia has become a serious disease in the world. As a common metabolic abnormality disease, the prevalence of hyperglycaemia in China was continually increased and ranked first in the world^(1,2)^. Hyperglycaemia is a well-known major factor of metabolic syndrome, which gives rise to a huge burden on the healthcare system^(3)^. Hence, several methods have been used to reduce the risk of hyperglycaemia, such as exercise, lifestyle changes and dietary pattern improvement^(4)^. Moreover, medical nutrition therapy has become a focal treatment for hyperglycaemia, which aims to find an optimal macronutrient composition and energy intake^(5)^. However, unstable protein ingestion is not recommended for subjects due to malnutrition or nephropathy^(6)^. For this reason, the macronutrient composition was always regulated by carbohydrates and fat. In China, the average proportions of carbohydrates and fat intake among adults were higher than in the Western populations, although the carbohydrate intake has reduced in recent years^(7,8)^. Several studies have reported that the proportions of carbohydrates and fat were associated with chronic disease^(9,10)^. However, there is a lack of research on the correlation between this proportion and hyperglycaemia. Therefore, we conducted this study and aimed to address the relationship between the proportions of carbohydrates and fat and hyperglycaemia in the Chinese population.

## Materials and methods

### Study design and population

The China Health and Nutrition Survey, an ongoing cohort research, provided the data that we used. The China Health and Nutrition Survey, which is a longitudinal research, tries to spot changes in geographic areas, public services, economic growth, nutritional status and health indicators. Since the beginning of the China Health and Nutrition Survey in 1989, the population has been monitored every 2–4 years. A sample of over 7200 homes, encompassing more than 30 000 people in 15 provinces, was obtained. An earlier cohort profile explains in full the design and sampling procedures of the China Health and Nutrition Survey^(11)^.

**Fig. 1:**
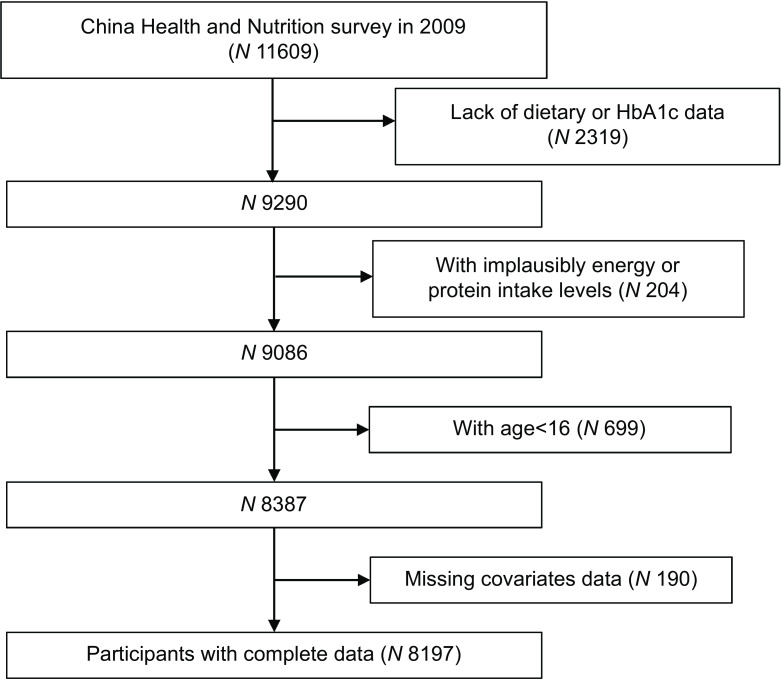
Flow chart of the sample selection methods in each step

The data we used were obtained in 2009. The data mainly contain dietary intake, blood indicators, physical measurements and lifestyle information. After excluding participants with missing dietary data, BMI, blood pressure and blood biomarker messages, implausibly low or high energy intake levels (<500 or >5000 kcal), abnormal proportions of energy from protein (<5 % or >20 %) or age under 16 years, 8197 individuals were included in the final analysis. The participant selection process is described in Fig. 1.

### Dietary data collection

The dietary intake of participants was recorded by the trained interviewer, through a 3-d 24-h recall method at the individual level, and a food inventory was performed at the household level over the same 3-d period^(12)^. The household food consumption was calculated by measuring the changes in the inventory from the beginning to the end of the survey. The individual average total energy and nutrient intakes (carbohydrates, fats and proteins) were calculated based on the China food composition tables^(13)^.

### Carbohydrate and fat proportion groups

When the ratio of protein intake is normal, a high level of carbohydrates is inevitably accompanied by a low level of fat, and vice versa. Therefore, we divided the subjects into four groups based on their proportion of carbohydrates: low-carbohydrate and high-fat group (LCHF), when the proportions of energy from carbohydrates < 45 %; the medium proportion of carbohydrate and fat group (MPCF), 60 % ≥ proportions of energy from carbohydrate ≥ 45 %; high-carbohydrate and low-fat group (HCLF), 75 % > proportions of energy from carbohydrate > 60 %; and very-high-carbohydrate and low-fat group (VHCLF), proportions of energy from carbohydrate ≥ 75 %.

### Hyperglycaemia

The blood samples of participants were collected and assayed in 2009, and follow-up blood data have not been made publicly available. Blood samples were collected in three tubes, a total of 12 ml of blood for individuals greater than 7 years old. Fasting blood glucose was measured through the GOD-PAP by Hitachi 7600 and tested in Beijing Central Laboratory, and glycated Hb was tested by hlc-723G7 in the provincial laboratory. In addition, subjects were asked to report their diabetes status and treatment ways. Thus, in our study, hyperglycaemia was defined as glycated Hb ≥ 5·7 %, or fasting blood glucose ≥ 6·1 mmol/l, or self-reported diabetes or taking any anti-hyperglycaemic therapy^(14)^.

### Other variables

The participants were divided into groups based on their alcohol-drinking status, tea-drinking status and smoking status (Yes/No). Location was recorded as urban or rural. Overweight was defined as BMI ≥ 24·0 kg/m^2^, and subjects were divided into two levels (Yes/No)^(15)^. People aged below 45 years were defined as young, and we divided participants into two groups (Yes/No). The blood pressure was calculated by the mean of three measurements. Individuals were diagnosed with hypertension when diastolic blood pressure (DBP) ≥ 90 mmHg or systolic blood pressure (SBP) ≥ 140 mmHg or self-reported hypertension or taking anti-hypertension drugs. Education was divided into four levels: none, primary school graduate, middle school graduate and a college degree, and a college degree was defined as high education level. Energy intake was divided into the following four levels: less than 1400, 1400∼2000, 2000∼2600 kcal and more than 2600 kcal.

### Statistical analysis

The data analysis software package SAS 9·4 (SAS Institute Inc.) was used for the data analysis. The mean and sd are used to describe the continuous variables (e.g. age). When the data were normally distributed, we used ANOVA to detect the differences in the four groups. The *χ* ^2^ test was used to detect the differences in sex, location, education levels, tea drinking, smoking and hypertension. The multivariable logistic regression was used to calculate the OR and 95 % CI of hyperglycaemia in each group, and the MPCF diet intake group was used as the reference. The adjusted variables included age, BMI, gender, location, education, total energy, hypertension, smoking, alcohol drinking and tea-drinking status. The significance level was set at 0·05 (two-sided).

## Results

### Characteristics of the participants

The characteristics of the study individuals are presented in Table 1. There were 8197 participants in this study, and the overall prevalence of hyperglycaemia was 38·4 %. Among the total participants, 1345 subjects had an LCHF diet, 3951 individuals had an MPCF diet, 2660 participants had an HCLF diet, and 241 people had the VHCLF diet. Compared with individuals in other groups, those with an LCHF diet were older and more likely to live in urban, be highly educated, have higher energy intake and included a greater proportion of subjects who drank alcohol and tea and smoked. Furthermore, the participants with a VHCLF diet were more likely to live in rural, be lowly educated, have a lower energy intake and include a smaller ratio of individuals who drank alcohol and tea and smoked.

**Table 1:** Baseline characteristics of the individuals in the four carbohydrate-fat proportion groups

Factor	LCHF		MPCF		HCLF		VHCLF		*P*
	*n*	%	*n*	%	*n*	%	*n*	%	
Subjects (*n*, %)	1345	16·4	3951	48·2	2660	32·5	241	2·9	
Female (*n*, %)	677	50·3	2148	54·4	1413	53·1	133	55·2	0·074
Age (years)	51·9	15·5	49·9	15·3	49·2	15·2	50·0	14·9	0·001
Urban (*n*, %)	749	55·7	1304	33·0	553	20·8	38	15·8	0·001
High-level education (*n*, %)	221	16·4	545	13·8	187	7·0	12	5·0	0·001
BMI (kg/m^2^)	23·6	3·5	23·3	3·5	23·1	3·5	23·5	3·4	0·001
Energy (kcal)	2195·0	646·6	2146·6	614·6	2083·6	619·6	1870·6	611·3	0·001
SBP (mmHg)	125·5	19·2	124·8	19·0	124·3	19·5	123·9	18·0	0·223
DBP (mmHg)	80·2	11·3	80·7	11·4	80·4	11·6	80·1	11·0	0·555
HbA1c (%)	5·7	1·0	5·6	0·9	5·6	1·0	5·7	0·8	0·005
Hypertension (*n*, %)	436	32·4	1227	31·1	769	28·9	72	29·9	0·106
Hyperglycaemia (*n*,%)	551	50·0	1460	37·0	1014	38·1	119	49·4	0·001
Smoked (*n*, %)	454	33·8	1169	29·6	831	31·2	65	27·0	0·018
Tea (*n*, %)	647	48·1	1412	35·7	749	28·2	50	20·8	0·001
Alcohol (*n*, %)	531	39·5	1278	32·4	798	30·0	51	21·2	0·001

LCHF, low carbohydrate and high fat; MPCF, medium proportion of carbohydrate and fat group; HCLF, high carbohydrate and low fat; VHCLF, very high carbohydrate and low fat; HbA1c, glycated Hb.

### Risk of hyperglycaemia by different carbohydrate-fat proportions

The OR (95 % CI) of the association between hyperglycaemia and carbohydrate-fat proportion is summarised in Table 2. After adjusting for age, sex, BMI, location, energy, education alcohol, smoking, tea drinking and hypertension, subjects with the HCLF diet were significantly associated with an increased risk of hyperglycaemia (OR: 1·142; 95 % CI: 1·022, 1·276), when compared with the individuals with the MPCF diet. Meanwhile, people with the VHCLF diet had a higher risk of hyperglycaemia (OR: 1·829; 95 % CI: 1·377, 2·429). In contrast, the association between participants with an LCHF diet and hyperglycaemia was reversed to NS (OR: 1·082; 95 % CI: 0·942, 1·243), after adjusting a series of above confounding factors.

**Table 2:** OR (95 % CI) of the association between hyperglycaemia and carbohydrate-fat proportion

	Groups						
	LCHF	95 % CI	MPCF	HCLF	95 % CI	VHCLF	95 % CI
Model 1	1·184	1·043, 1·344	1	1·051	0·950, 1·163	1·664	1·282, 2·160
Model 2	1·067	0·931, 1·222	1	1·131	1·015, 1·261	1·774	1·341, 2·346
Model 3	1·083	0·943, 1·244	1	1·137	1·018, 1·270	1·805	1·361, 2·395
Model 4	1·082	0·942, 1·243	1	1·142	1·022, 1·276	1·829	1·377, 2·429

LCHF, low carbohydrate and high fat; MPCF, medium proportion of carbohydrate and fat group; HCLF, high carbohydrate and low fat; VHCLF, very high carbohydrate and low fat.

Model 1 adjusted no variable.

Model 2 adjusted age, sex and BMI.

Model 3 adjusted age, sex, BMI, location, energy and education.

Model 4 adjusted age, sex, BMI, location, energy, education, alcohol, smoking, tea drinking and hypertension.

### Influence of different energy intake levels on the association between carbohydrate-fat proportion and hyperglycaemia

After adjusting for age, sex, BMI, location, education alcohol, smoking, tea drinking and hypertension, we estimated the associations of carbohydrate-fat proportion with hyperglycaemia at different energy intake levels. As shown in Table 3, when compared with the individuals with an MPCF diet, those with the HCLF diet had a higher risk of hyperglycaemia in the low energy intake level (OR: 1·427; 95 % CI: 1·029, 1·981). Furthermore, people with a VHCLF diet had a higher risk of hyperglycaemia in the low and middle energy intake levels, the OR (95 % CI) was 2·152 (1·153, 4·020), 1·795 (1·111, 2·901) and 2·068 (1·231, 3·472), respectively. Meanwhile, we have not observed these significant associations in the high energy intake level.

**Table 3.** OR (95 % CI) of the association between carbohydrate-fat proportion and hyperglycaemia in different energy levels

Energy levels (kcal)	Groups						
	LCHF	95 % CI	MPCF	HCLF	95 % CI	VHCLF	95 % CI
E < 1400	1·247	0·798, 1·950	1	1·427	1·029, 1·981	2·152	1·153, 4·020
2000 > E ≥ 1400	1·168	0·908, 1·503	1	1·130	0·932, 1·370	1·795	1·111, 2·901
2600 > E ≥ 2000	0·943	0·745, 1·195	1	1·056	0·872, 1·278	2·068	1·231, 3·472
E ≥ 2600	1·143	0·862, 1·514	1	1·176	0·924, 1·497	1·343	0·609, 2·963

LCHF, low carbohydrate and high fat; MPCF, medium proportion of carbohydrate and fat group; HCLF, high carbohydrate and low fat; VHCLF, very high carbohydrate and low fat.

### Subgroup analyses of the association between carbohydrate-fat proportion and hyperglycaemia

**Fig. 2:**
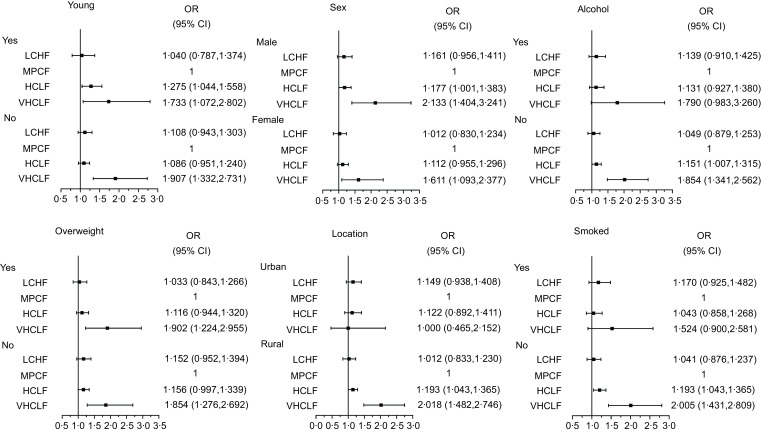
Subgroup analyses of the association between carbohydrate-fat proportion and hyperglycaemia (LCHF, low carbohydrate and high fat; MPCF, medium proportion of carbohydrate and fat group; HCLF, high carbohydrate and low fat; VHCLF, very high carbohydrate and low fat)

We further evaluated the associations of carbohydrate-fat proportion with hyperglycaemia in each subgroup. As shown in Fig. 2, the participants who intake an HCLF and VHCLF diets were significantly associated with a high risk of hyperglycaemia in the most of subgroups, when compared with those who had an MPCF diet, especially the people who were living in rural (OR: 1·138; 95 % CI: 1·003, 1·292 and OR: 2·018; 95 % CI: 1·482, 2·746, respectively).

## Discussion

By using data from a nationally, large sample size cross-sectional cohort in China, we observed that there was a significant association between the proportion of carbohydrate-fat intake and hyperglycaemia in adults. In detail, the individuals with an HCLF and VHCLF diet were significantly associated with a higher risk of hyperglycaemia. Furthermore, people with a VHCLF diet were significantly associated with a higher risk of hyperglycaemia in the major energy levels and social characteristics subgroup.

To the best of our knowledge, this is the first study to explore the association of different proportions of carbohydrate-fat intake with hyperglycaemia among nationally representative Chinese adults. To date, lots of studies have investigated the association between the different proportions of carbohydrate-fat intake and the risk of diabetes and CVD^(16–18)^. However, there are no consistent conclusions based on these associations. For instance, one research demonstrated that a high-fat and low-carbohydrate diet score was associated with the high incidence of type 2 diabetes in the Chinese population^(18)^. On the contrary, another study in Japan indicated that a low-carbohydrate diet was associated with a decreased risk of type 2 diabetes in women^(17)^. Additionally, two studies about the Hawaii diet and Okinawa diet models described that the highly complex carbohydrate diet and a healthy fat profile can decrease the risk of CVD and blood glucose values^(19,20)^. Nevertheless, it is unclear whether the effect of these special diet models on people is due to the proportions of carbohydrates and fats or their mineral content. And Chinese people always intake refined cereals as their staple food which may be different from the carbohydrates in this research. In our study, individuals with an HCLF and VHCLF diet were significantly associated with a higher risk of hyperglycaemia. It was consistent with one previous animal experiment, which presented that a high-carbohydrate diet intake can increase postprandial glycaemia in healthy cats when compared with diets high in fat or protein^(21)^. Meanwhile, we found there was no significant association between the LCHF diet and hyperglycaemia in our study. Similarly, one research that included 1018 pregnant women shows these associations were NS after adjustment for covariates^(22)^. In the past, the LCHF diet was usually used for weight loss and treatment of epilepsy, though there is a lack of data supporting long-term efficacy and safety^(23)^.

Not only that, we explored these associations in various energy intake levers. We found that people with the HCLF and VHCLF diet had a higher risk of hyperglycaemia in the low and middle energy intake levels and these associations were NS in the highest energy levels. Our results indicated that when the total energy intake was appropriate, people needed to decrease the proportions of carbohydrate-fat, which may be conducive to reducing the risk of hyperglycaemia. Moreover, our results also show that the participants who intake an HCLF and VHCLF diets were significantly associated with a high risk of hyperglycaemia in the majority of subgroups, especially those who live in rural. One previous survey reported that people who live in rural areas have a higher prevalence and lower awareness rate about hyperglycaemia^(24)^. Meanwhile, rural dwellers may lack sufficient health knowledge and medical support, which also could strengthen the associations between the different proportions of carbohydrate-fat intake and the risk of hyperglycaemia.

Currently, the underlying mechanisms have not been clearly elucidated, and several possible illustrations have been proposed. First, the most of carbohydrates in HCLF diets in China were composed of refined grain which has a high glycaemic index. However, high glycaemic index foods can elicit higher glycaemic and insulinemic responses and promote insulin resistance through β-cell exhaustion^(25)^. Meanwhile, insulin resistance can increase the risk of adverse cardiovascular events, and then these factors further interact and increase the levels of blood glucose^(26)^. Second, the HCLF diet could upregulate the markers of inflammation and oxidative stress by generating reactive oxygen species through several pathways, which also lead to insulin and impaired insulin secretion^(27,28)^. Third, a previous study reported that people who took an HCLF diet had lower levels of serum total adiponectin^(29)^. Nevertheless, the lack of adiponectin could contribute to glucose intolerance and hyperglycaemia^(30)^. In turn, abundant adiponectin can reduce the burden of liver ceramide and improve the effect of hepatic insulin^(31)^. We think these illustrations may explain how an HCLF diet affects hyperglycaemia.

The strengths of this study include the large sample size of participants in the real world. Furthermore, we first clearly show the association between the different proportions of carbohydrates and fat and hyperglycaemia. We found that individuals who intake an HCLF and VHCLF diet were significantly associated with a higher risk of hyperglycaemia. However, limitations also exist in this study. First, we calculated the individual intake of oil based on consumption only at home, which may cause an underestimation of the intake of fat. Second, we did not differentiate between different types of carbohydrates and fats, which may have unique effects on hyperglycaemia. Third, we cannot eliminate all unmeasured or residual confounding factors, which may affect the associations observed in this study, even though we have adjusted for many confounders in the analysis step.

### Conclusion

In conclusion, our study indicated that people with the HCLF and VHCLF diets were significantly associated with a higher risk of hyperglycaemia. And these results could help people comprehend their dietary structure and adjust appropriately to prevent and treat hyperglycaemia.
